# Introduction: 35 years on

**DOI:** 10.1101/gad.350546.123

**Published:** 2023-01-01

**Authors:** John R. Inglis

**Affiliations:** Cold Spring Harbor Laboratory Press, Cold Spring Harbor, New York 11797, USA

This January 2023 special issue of *Genes & Development* is an appreciation of the work of the journal's Editor, Terri Grodzicker, who stepped down in December 2022 after 35 years of distinguished service. It consists of personal essays specially commissioned from 28 prominent scientists, to whom we are grateful for accepting the invitation and bringing such care and thoughtfulness to the task. They were chosen as representatives of the large group of scientists worldwide who have supported *G&D* by publishing significant papers, acting as peer reviewers, and offering advice both informally and as advisory board members. In the course of three decades, they got to know Terri and the journal extremely well.

We knew that our request to write so personally about this relationship was unusual and perhaps challenging. But we hoped that for this assignment, the authors would feel free of the customary conventions of scholarly communication, and readers would see the articles in that light. We are also grateful to Carol Prives, Rudi Grosschedl, and Robert Tjian for their willingness to guest edit this issue, making suggestions for contributors and reviewing all the articles received.

We placed no constraints on the nature of the articles we requested, so they vary widely in style and approach. Some reflect on the circumstances in which *G&D* began. Some contain pungent comments on the state of science publishing today. But all the essays acknowledge the huge influence journal editors—and their decisions—have on the progression of science and the development of scientists’ careers. And all express much admiration for Terri—an innovative bench scientist who in 1989 took on a daunting role as full-time editor of an ambitious journal launched by an academic institution with no journal publishing experience into a competitive arena dominated by commercial publications with mostly male editors.

The writers emphasize the special qualities Terri brought to her chosen task: wide interests, exceptional scientific acumen, willingness to listen to experts, and the courage to be decisive. These skills had a vital part to play in *G&D*’s improbably rapid rise into the top ranks of biomedical research journals. In the decades to come, Terri would accept more than 7800 research and review articles for publication. These have been cited 1.5 million times and include many papers with several thousand individual citations that are classics in the literature. Her legacy is the widespread respect for *G&D* in the international research community and its reputation for being fair and quick in its editorial decisions and publishing first-class science. It's an achievement we intend to build on.

Even great editors need great teams. During Terri's tenure, *G&D* has been fortunate to have had 10 talented editorial staff members; several long-serving, dedicated production editors; and highly professional support from colleagues in marketing, advertising sales, and finance. For the past 7 years, Laureen Connell has had an essential role as the journal's Senior Editor and will now serve as Executive Editor, in concert with an evolving group of academic editors. And for the past 21 years, Bibi Garite has been at the heart of the journal's administration, providing firm and friendly guidance for reviewers, board members, and authors, playing a vital part in the assembly of issues as she did for this one.

We hope readers will enjoy this collective appreciation of Terri Grodzicker's remarkable contribution to the advance of biomedical science.

